# Herpes Zoster of the Larynx: A Diagnostic and Therapeutic Challenge

**DOI:** 10.7759/cureus.100666

**Published:** 2026-01-03

**Authors:** Noémi Nogueira, Teresa Bernardo, Simão Bessa, Joana Ferreira, Nuno Lousan

**Affiliations:** 1 Department of Otorhinolaryngology, Head and Neck Surgery, Unidade Local de Saúde do Tâmega e Sousa, Porto, PRT

**Keywords:** dysphagia, herpes zoster, laryngitis, odynophagia, varicella-zoster virus

## Abstract

Herpes zoster of the larynx is an exceptionally rare condition that may mimic more common causes of laryngitis. Reactivation of varicella-zoster virus in the vagus nerve can lead to odynophagia, dysphonia, and dysphagia, often without the typical cutaneous rash. Early recognition is essential, as delayed diagnosis may result in serious complications, including postherpetic neuralgia, persistent dysphagia, chronic voice impairment, and even laryngeal paralysis. We describe a 66-year-old woman on long-term low-dose corticosteroid therapy who presented with acute odynophagia, dysphagia, dysphonia, and fever. Flexible nasopharyngolaryngoscopy revealed supraglottic vesicles localized to the right hemilarynx, extending to the epiglottis and arytenoid region, with ipsilateral sensory loss. Serological testing confirmed varicella-zoster virus infection. The patient was treated with valacyclovir and gabapentin, with resolution of odynophagia and mucosal lesions at one month, though mild dysphagia persisted. Laryngeal herpes zoster represents a diagnostic challenge due to its rarity, overlapping symptoms with other forms of laryngitis, and the brief window of opportunity in which vesicular lesions are visible. The scarcity of reported cases underscores the importance of maintaining a high index of suspicion, particularly in immunosuppressed patients. Early antiviral therapy is crucial to reduce complications, while endoscopic evaluation plays a key role in timely recognition.

## Introduction

Varicella-zoster virus (VZV), responsible for chickenpox, establishes latency in cranial and dorsal root ganglia after primary infection and may reactivate as Herpes Zoster (HZ) when cell-mediated immunity wanes, particularly with aging or immunosuppression [1,2]. HZ typically presents as painful vesicular eruptions in a dermatomal distribution, but head and neck reactivation can also manifest with cranial neuropathies and mucosal disease [2]. Laryngeal involvement, also known as Herpes Zoster laryngitis (HZL), reflects reactivation of the vagus nerve and is rare and likely underdiagnosed, in part because symptoms overlap with viral laryngitis, and vesicular lesions are short-lived [3,4]. The most common symptoms include severe odynophagia and dysphonia, and occasionally sensory and motor dysfunction such as reduced laryngeal reflexes and impaired swallowing [3]. Recognition is further complicated when cutaneous lesions are absent (*zoster sine herpete*), in which case laboratory confirmation with polymerase chain reaction (PCR) from mucosal lesions or saliva or, in selected scenarios, cerebrospinal fluid testing for VZV DNA or intrathecal anti-VZV immunoglobulin (Ig)G may be required [5].

Endoscopic visualization of unilateral supraglottic vesicles with a clear midline stop and ipsilateral sensory changes is highly suggestive of HZL and helps define the narrow diagnostic window [4]. Prompt antiviral therapy, ideally within 72 hours of symptom onset, shortens the acute course and reduces pain. Treatment beyond this window remains reasonable if new lesions are evolving or if neurological complications are present [2]. Early diagnosis is essential to prevent complications such as postherpetic neuralgia and persistent voice or swallowing dysfunction [2,3]. Historical and contemporary reports highlight the narrow diagnostic/therapeutic window and the risk of lingering dysphagia or voice dysfunction even after mucosal healing [3,4,6-9]. When diagnosis and therapy are delayed, severe outcomes, including vocal fold paralysis and marked dysphagia, have been reported [7,10,11]. Against this background, we present a unilateral, rash-negative HZL in an immunosuppressed patient, highlighting the roles of early endoscopy, timely antivirals, and structured functional follow-up.

## Case presentation

**Figure 1:**
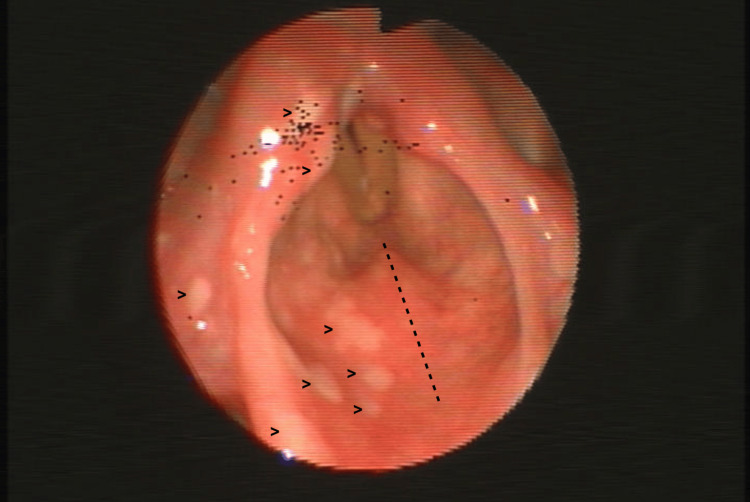
Flexible nasopharyngolaryngoscopy of the larynx Supraglottic vesicles (arrowheads) confined to the right hemilarynx, with a clear midline stop (dotted line).

**Figure 2:**
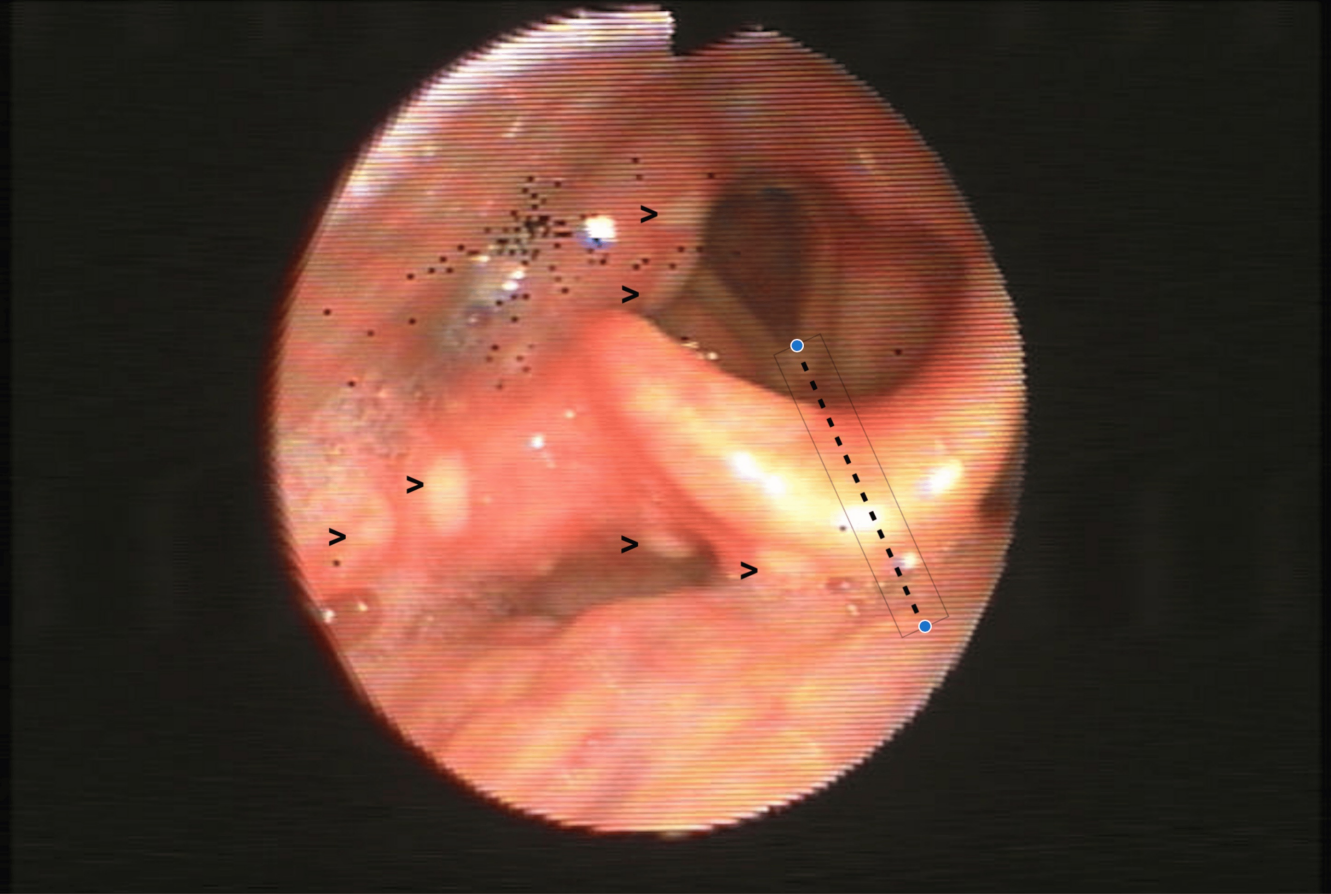
Flexible nasopharyngolaryngoscopy of lesion extension Vesicles (arrowheads) extending to the right glossoepiglottic and pharyngoepiglottic folds with ipsilateral arytenoid edema and salivary stasis. Lesions terminate at a clear midline boundary (dotted line), with contralateral sparing.

A 66-year-old woman presented to the emergency department with three days of intense odynophagia, dysphagia to solids and liquids, dysphonia and fever. She had been taking low-dose prednisolone daily for four months for suspected rheumatoid arthritis. She denied cutaneous rash or otalgia. Examination of the oral cavity and oropharynx revealed no abnormalities. Indirect laryngoscopy, complemented by flexible nasopharyngolaryngoscopy, showed clusters of supraglottic vesicles confined to the right hemilarynx with a clear midline stop (Figure 1), affecting both surfaces of the epiglottis and extending to the right glossoepiglottic and pharyngoepiglottic folds with ipsilateral arytenoid edema (Figure 2). Vocal fold mobility was preserved. Laryngeal penetration of saliva was observed on endoscopic examination. Gentle contact of the fibroscope with the ipsilateral aryepiglottic fold revealed decreased sensation. During a supervised swallow of liquids, a cough reflex was consistently elicited. 

Routine vital signs and laboratory results obtained at presentation are summarized in Table 1.

**Table 1:** Vitals and laboratory results at presentation WBC: white blood cells; VZV: varicella-zoster virus, Ig: immunoglobulin. Units and reference intervals are as reported by the institutional laboratory.

Parameter	Result	Reference range	Units
Blood pressure	128/77	—	mmHg
Heart rate	88	60-100	beats/min
Temperature	38.6	<37.5	°C
Oxygen saturation (room air)	99	>92	%
Hemoglobin	14.4	12.0-15.0	g/dL
Platelets	225	150.0-400.0	x10^3^/uL
WBC	6.80	4.5-11.0	×10^3^/uL
Neutrophils	3.98	2.0-7.5	×10^3^/uL
Lymphocytes	1.84	1.5-4.0	×10^3^/uL
C-reactive protein	7	<5.0	mg/L
Creatinine	0.78	0.66-1.09	mg/dL
VZV IgM	Positive	—	—
VZV IgG	Positive	—	—

On further history, the patient reported a prior episode of herpes zoster in the right C4 dermatome 20 years earlier. Serologic testing supported recent varicella-zoster virus reactivation, with positive IgM and IgG in the appropriate clinical context. In view of the unilateral vesicular lesions with a midline boundary, ipsilateral sensory deficit, immunosuppression due to chronic steroids, and supportive serology, herpes zoster laryngitis was favored over nonspecific viral laryngitis, candidiasis, contact ulcer, or early neoplasia. Molecular confirmation by polymerase chain reaction testing was considered but not pursued, given the characteristic endoscopic findings, compatible serology, and expected therapeutic response.

Antiviral therapy was initiated at presentation with valacyclovir 1,000 mg orally three times daily for seven days. Neuropathic pain was managed with gabapentin, titrated to 300 mg three times daily as tolerated, and the patient was advised to follow a soft, cold diet with analgesia as needed. By day seven, she reported a meaningful reduction in throat pain and improved oral intake. At the 30-day follow-up visit, odynophagia and mucosal vesicles had completely resolved, while mild dysphagia persisted. She remains under follow-up in the otolaryngology clinic for ongoing monitoring of swallowing and voice function, with gradual improvement in dysphagia. She was subsequently referred to the infectious diseases clinic after what was considered a second episode of Herpes Zoster. Given her age and recent immunosuppression, recombinant zoster vaccination was prescribed as a two-dose series (0 and three months) to reduce the risk of recurrence and related complications. No adverse events related to the vaccine, valacyclovir, or gabapentin were observed or reported during treatment or follow-up.

## Discussion

HZL is an uncommon manifestation of VZV reactivation that frequently overlaps clinically with nonspecific viral laryngitis, leading to missed or delayed diagnoses [3,4]. When present, a unilateral distribution of vesicles with a clear midline stop, together with ipsilateral sensory changes, is highly suggestive and should prompt consideration of HZL. The diagnostic window is narrow because mucosal lesions are short-lived; therefore, timely endoscopic evaluation during the symptomatic period is pivotal to avoid missed or late diagnoses [4]. Atypical reactivation without a cutaneous rash (*zoster sine herpete*) further complicates recognition. In such scenarios, laboratory confirmation is warranted. Polymerase chain reaction (PCR) from mucosal lesions or saliva offers rapid, sensitive confirmation. In the presence of neurological involvement or diagnostic uncertainty, cerebrospinal fluid testing for VZV DNA or intrathecal anti-VZV IgG may be decisive [5,6]. These strategies are particularly relevant in older or immunocompromised patients, who are at increased risk of reactivation and sequelae [1,2].

Management should prioritize early antiviral therapy. Oral valacyclovir, famciclovir, or acyclovir, initiated ideally within 72 hours of symptom onset, shortens the acute course and reduces pain. Treatment beyond this window remains reasonable if new lesions are evolving or if neurological complications are present [2]. Neuropathic pain may require adjunctive agents such as gabapentin or tricyclic antidepressants, and persistent dysphagia or dysphonia may necessitate structured follow-up and supportive care [2]. The role of systemic corticosteroids is controversial. At the same time, they may hasten short-term symptomatic improvement in selected zoster presentations, but they do not reliably prevent postherpetic neuralgia and should be used cautiously, especially in patients already receiving steroids [2].

Available series suggest that HZL is underdiagnosed and associated with older age, vascular comorbidity, and a higher likelihood of multiple cranial nerve involvement than in other head and neck zoster presentations [3]. When diagnosis and therapy are delayed, severe complications have been described in related cranial neuropathies, including vocal fold paralysis and marked dysphagia, underscoring the clinical importance of prompt recognition and treatment [7]. In the present case, early endoscopic identification of unilateral supraglottic vesicles and prompt antiviral therapy coincided with resolution of pain and mucosal disease, although mild dysphagia persisted, consistent with the recognized risk of lingering neurosensory dysfunction in this entity [2-4]. Beyond acute management, post-episode recombinant zoster vaccination is recommended for adults ≥50 years and for immunocompromised adults. It should be administered once the acute illness has fully resolved, and a two-dose series (0 and two to six months, or one to two months in immunocompromised adults) is advised [12-14]. This preventive step is pertinent in patients with recurrent zoster or recent immunosuppression and may mitigate future episodes and sequelae [12,13].

## Conclusions

HZL is a rare cause of laryngitis that requires a high index of suspicion. It should be considered in patients with severe odynophagia and unilateral supraglottic lesions, particularly in the context of immunosuppression. Early endoscopic evaluation during the brief vesicular phase and prompt antiviral therapy are central to limiting complications and long-term voice and swallowing sequelae. Awareness of rash-negative presentations and judicious use of confirmatory testing can prevent diagnostic delay and improve outcomes. The limited literature underscores the need for further studies addressing clinical course, optimal management, and long-term outcomes.
